# Metabolic syndrome and lung function in Korean children and adolescents: A cross-sectional study

**DOI:** 10.1038/s41598-019-51968-2

**Published:** 2019-10-30

**Authors:** Minji Kim, Seoheui Choi, Soo-Han Choi, Seon-Hee Shin, Sung Koo Kim, Young Suk Shim, You Hoon Jeon

**Affiliations:** 1 Department of Pediatrics, Hallym University, Dongtan Sacred Heart Hospital, Hwaseong, Gyeonggi-do, Korea; 2 Allergy and Clinical Immunology Research center, Hallym University College of Medicine, Chuncheon, Korea

**Keywords:** Endocrinology, Metabolic syndrome, Risk factors, Obesity, Risk factors

## Abstract

This study aimed to investigate whether obesity and metabolic syndrome (MetS) are associated with pulmonary function in Korean children and adolescents. Data from the 2009–2011 Korea National Health and Nutrition Examination Survey which is cross-sectional, nationwide, and representative survey were used. Adjusted regression analysis was performed to evaluate the association of obesity and MetS with lung function in children and adolescents. A total of 763 children and adolescents aged 10–18 years were evaluated. We found no significant difference in FEV_1_% predicted, FVC% predicted, and FEV_1_/FVC ratio among the obesity groups. Subjects with MetS showed a significantly lower FEV_1_ predicted (91.54 ± 0.74% vs 94.64 ± 0.73%, P = 0.004), lower FVC% predicted (91.86 ± 0.63% vs 95.20 ± 0.63%, P < 0.001), and lower FEV_1_/FVC ratio (76.76 ± 0.43% vs 80.13 ± 0.43%, P < 0.001) than those without MetS. Elevated waist circumference (WC), systolic blood pressure, fasting glucose, and lower high-density lipoprotein cholesterol (HDL-C) were independently associated with lower FEV_1_/FVC ratio (all P < 0.05, respectively). Among MetS components, increased WC was the most important factor influencing lower FEV_1_/FVC ratio. In conclusion, lung function in MetS patients was significantly lower, and the MetS component was independently associated.

## Introduction

Obesity is a worldwide public health issue with increasing incidence^1^. In the US, approximately 35% of adults and 17% of children and adolescents are obese^2^. The prevalence of overweight or obesity in Korean adolescents has significantly increased from 16.4% in 1998 to 24.2% in 2005^3^. Metabolic syndrome (MetS) is a common metabolic disorder that can result from obesity^4^. MetS is defined as abdominal obesity, hypertension, dyslipidemia, and glucose intolerance^5^. In the US, roughly more than 20% of the population and 60% of obese people have MetS^6^. In Korea, MetS is prevalent in 5.7% of adolescents and in 30.5% of adults^7,8^. Research interest to MetS-related diseases has been increasing owing to the increasing incidence of obesity and MetS.

Overweight and obesity are associated with lower lung function in adults, but whether such association also exists in children is unclear^9,10^. Studies have reported that obesity in children and adolescents is associated with a high forced expiratory volume in the 1st second (FEV_1_) and forced vital capacity (FVC) and a lower FEV_1_/FVC^10^. However, one study reported that obese non-asthma children showed lower residual capacity, residual volume, and expiratory reserve volume than their non-obese counterparts^11^. Further, other studies have reported no significant association between obesity and pulmonary function^12^.

The association between MetS and pulmonary function is also controversial. One cross-sectional study in France reported that MetS is associated with lower FEV_1_ or FVC^13^, and another study reported that MetS is associated with significant restrictive pattern of lung function^14^. Although increasing evidence indicates that MetS may be associated with lung disease, the mechanisms behind this association are complex and are yet to be clearly understood. Moreover, although the respiratory system of children is different from that of adults, studies about the association between lung function and MetS in children and adolescent are scarce.

Therefore, the purpose of this study was to analyze the association of obesity and MetS with pulmonary function to help understand the mechanism by which obesity or MetS influences pulmonary function in children and adolescents and provide a basis for establishing related health policies in the future.

## Results

### Clinical characteristics of the study participants

Of the 1,958 subjects aged 10–18 years identified, those who had no data for anthropometrical information (n = 160), BMI and blood pressure (n = 317), and pulmonary function test (n = 717) were excluded. Thus, 763 children and adolescents aged 10–18 years (414 males and 349 females) were included in this study. The clinical characteristics of the study population are summarized in Table 1. The mean age was 13.61 ± 0.09 years. In total, 235 (30.8%) and 224 (29.4%) participants were overweight and obese, respectively. Compared with the normal weight group, the overweight and obesity groups were younger (P < 0.001), more likely to be female (P < 0.001), and taller (P < 0.001). Blood pressure (P < 0.001), serum glucose (P = 0.043), total cholesterol (P = 0.001), TG (P = 0.009), and LDL-C (P = 0.001) were significantly different between the three groups, whereas no significant differences in alcohol drinking, smoking, physical activity, household income, the prevalence of diabetes, atopic dermatitis, and asthma were noted.

**Table 1:** Clinical characteristics of the study population according to the obesity in Korean boys and girls aged 10–18 years (n = 763).

	Total	Normal weight	Overweight	Obesity	*P*
	(n = 763)	(n = 304)	(n = 235)	(n = 224)	
Male (%)	414 (54.3%)	197 (64.8%)	134 (57.0%)	83 (37.1%)	<0.001
Age (years)	13.61 ± 0.09	14.61 ± 0.13	13.51 ± 0.15	12.36 ± 0.14	<0.001
Height SDS	0.88 ± 0.08	0.10 ± 0.11	1.03 ± 0.13	1.79 ± 0.13	<0.001
Weight SDS	1.28 ± 0.04	0.33 ± 0.06	1.48 ± 0.05	2.36 ± 0.05	<0.001
BMI SDS	1.18 ± 0.03	0.43 ± 0.03	1.33 ± 0.01	2.05 ± 0.02	<0.001
WC SDS	1.27 ± 0.03	0.59 ± 0.04	1.43 ± 0.03	2.04 ± 0.03	<0.001
SBP (mmHg)	123.5 ± 0.61	120.67 ± 0.96	123.28 ± 1.08	127.58 ± 1.11	<0.001
DBP (mmHg)	78.82 ± 0.38	76.70 ± 0.60	79.23 ± 0.65	81.26 ± 0.69	<0.001
Glucose (mg/dL)	99.25 ± 0.78	96.84 ± 1.12	100.66 ± 1.72	101.03 ± 1.22	0.043
T-C (mg/dL)	191.77 ± 1.36	185.88 ± 1.98	193.34 ± 2.44	198.13 ± 2.71	0.001
HDL-C (mg/dL)	47.96 ± 0.43	49.94 ± 0.71	47.66 ± 0.77	45.59 ± 0.74	<0.001
TG (mg/dL)	141.74 ± 3.89	128.28 ± 6.98	144.96 ± 6.68	156.62 ± 5.88	0.009
LDL-C (mg/dL)	116.69 ± 1.21	111.77 ± 1.73	117.77 ± 2.20	122.26 ± 2.46	0.001
Alcohol drinking (%)	321 (42.1%)	119 (39.1%)	108 (44.7%)	97 (43.3%)	0.394
Smoking (%)	141 (18.5%)	58 (19.1%)	40 (17.0%)	43 (19.2%)	0.786
Physical activity (%)	381 (49.9%)	139 (45.7%)	127 (54.0%)	115 (51.3%)	0.141
Household income (≤lowest quartile, %)	101 (13.2%)	48 (15.8%)	27 (11.5%)	6 (11.6%)	0.238
Residence (rural, %)	117 (15.3%)	48 (15.8%)	38 (16.2%)	31 (13.8%)	0.756
Hypertension (%)	199 (26.1%)	52 (17.1%)	62 (26.4%)	85 (37.9%)	<0.001
Dyslipidaemia (%)	101 (13.2%)	26 (8.6%)	37 (15.7%)	38 (17.0%)	0.007
Diabetes (%)	60 (7.9%)	19 (6.3%)	22 (9.4%)	19 (8.5%)	0.379
Asthma (%)	22 (2.9%)	5 (1.6%)	7 (3.0%)	10 (4.5%)	0.159
Atopic dermatitis (%)	13 (1.7%)	5 (1.6%)	2 (0.9%)	6 (2.7%)	0.317
FEV_1_ (%)	93.10 ± 0.51	91.71 ± 0.85	93.96 ± 0.85	94.06 ± 0.91	0.029
FVC (%)	93.54 ± 0.43	94.09 ± 0.71	94.49 ± 0.70	91.79 ± 0.81	0.084
FEV_1_/FVC (%)	78.73 ± 8.18	78.33 ± 9.30	78.04 ± 7.62	78.98 ± 7.04	0.439

SDS, standard deviation score; BMI, body mass index; WC, waist circumference; SBP, systolic blood pressure; DBP, diastolic blood pressure; T-C, total cholesterol; HDL-C, high-density lipoprotein cholesterol; TG, triglyceride; LDL-C, low-density lipoprotein cholesterol; FEV_1_, forced expiratory volume in one second; FVC, forced vital capacity; FVC forced vital capacity. Statistical significance was determined using a one-way ANOVA for continuous variables and a chi-square test for categorical variables (P < 0.05).

With respect to pulmonary function test, FEV_1_% predicted was significantly lower in the normal weight group than that in the obesity group, while FVC% predicted and FEV_1_/FVC ratio was not significantly different between these two groups.

The clinical characteristics of the study population according to the presence of MetS are summarized in Table 2. The overall prevalence of MetS was 49.8% (n = 380). Patients with the MetS were significantly younger (P < 0.001) and were less often male (P = 0.019) compared to participants without MetS. Metabolic components such as systolic blood pressure (SBP) (P < 0.001), diastolic blood pressure (DBP) (P < 0.001), waist circumference (WC) (P < 0.001), serum fasting glucose (P < 0.001), and serum triglyceride (TG) (P < 0.001) were significantly higher while HDL-C (P < 0.001) was significantly lower in participants with MetS than in those without MetS. Meanwhile, there was no significant difference in alcohol drinking, smoking, physical activity, household income, the presence of atopic dermatitis, and asthma between subjects with and without MetS. With respect to pulmonary function test, FEV_1_% predicted (91.73 ± 0.63 vs. 95.33 ± 0.57, P < 0.001) and FEV_1_/FVC ratio (76.83 ± 0.45 vs. 80.07 ± 0.45, P < 0.001) was significantly lower in those with MetS than those without MetS. Further, FVC% predicted tended to be lower in those with MetS (92.12 ± 0.75 vs. 94.06 ± 0.68, P = 0.055).

**Table 2:** Clinical characteristics of the study population according to the presence of metabolic syndrome (MetS) in Korean boys and girls aged 10–18 years (*n* = 763).

	Total	No MetS	MetS	*P*
	(n = 763)	(n = 383)	(n = 380)	
Male (%)	414	224 (58.5%)	190 (150.0%)	0.019
Age (years)	13.61 ± 0.09	13.85 ± 0.12	13.37 ± 0.13	<0.001
Height SDS	0.88 ± 0.08	0.53 ± 0.10	1.24 ± 0.11	<0.001
Weight SDS	1.28 ± 0.04	0.88 ± 0.06	1.69 ± 0.06	<0.001
BMI SDS	1.18 ± 0.03	0.87 ± 0.04	1.50 ± 0.03	<0.001
WC SDS	1.27 ± 0.03	0.85 ± 0.04	1.70 ± 0.03	<0.001
SBP (mmHg)	123.5 ± 0.61	117.84 ± 0.81	129.21 ± 0.82	<0.001
DBP (mmHg)	78.82 ± 0.38	76.11 ± 0.54	81.55 ± 0.48	<0.001
Glucose (mg/dL)	99.25 ± 0.78	92.90 ± 0.62	105.64 ± 1.37	<0.001
T-C (mg/dL)	191.77 ± 1.36	187.48 ± 1.73	196.09 ± 2.08	0.002
HDL-C (mg/dL)	47.96 ± 0.43	52.62 ± 0.58	43.26 ± 0.54	<0.001
TG (mg/dL)	141.74 ± 3.89	100.22 ± 3.21	183.58 ± 6.42	<0.001
LDL-C (mg/dL)	116.69 ± 1.21	115.10 ± 1.57	118.34 ± 1.86	0.182
Alcohol drinking (%)	321 (42.1%)	154 (40.2%)	167 (43.9%)	0.296
Smoking (%)	141 (18.5%)	66 (17.2%)	75 (19.7%)	0.373
Physical activity (%)	381 (49.9%)	185 (48.3%)	196 (51.6%)	0.366
Household income (≤lowest quartile, %)	101 (13.2%)	56 (14.6%)	45 (11.8%)	0.257
Residence (rural, %)	117 (15.3%)	59 (15.4%	58 (13.3%)	0.957
Hypertension (%)	199 (26.1%)	48 (12.5%)	151 (39.7%)	<0.001
Dyslipidaemia (%)	101 (13.2%)	6 (1.6%)	95 (25.0%)	<0.001
Diabetes (%)	60 (7.9%)	7 (1.8%)	53 (13.9%)	<0.001
Asthma (%)	22 (2.9%)	9 (2.3%)	13 (3.4%)	0.377
Atopic dermatitis (%)	13 (1.7%)	6 (1.6%)	7 (1.8%)	0.769
FEV_1_ (%)	93.10 ± 0.51	95.33 ± 0.57	91.73 ± 0.63	<0.001
FVC (%)	93.54 ± 0.43	94.06 ± 0.68	92.12 ± 0.75	0.055
FEV_1_/FVC (%)	78.43 ± 0.29	80.07 ± 0.45	76.83 ± 0.45	<0.001

The data are shown as the means ± SEs (standard errors).

MetS, metabolic syndrome; SDS, standard deviation score; BMI, body mass index; WC, waist circumference; SBP, systolic blood pressure; DBP, diastolic blood pressure; T-C, total cholesterol; HDL-C, high-density lipoprotein cholesterol; TG, triglyceride; LDL-C, low-density lipoprotein cholesterol; FEV_1_, forced expiratory volume in one second; FVC, forced vital capacity. Statistical significance was determined using independent *t*-test for continuous variables and a chi-square test for categorical variables (*P* < 0.05).

### Obesity and pulmonary function tests

After adjustment for age, sex, square of height (m^2^), household income, alcohol drinking, smoking, physical activity, and atopic dermatitis, there were no significant differences in FEV_1_ predicted (93.40 ± 1.21% vs. 93.77 ± 0.90%, 91.97 ± 1.47%, P = 0.529), FVC% predicted (94.36 ± 1.04% vs. 94.42 ± 0.77%, 91.51 ± 1.26%, P = 0.125), and FEV_1_/FVC ratio (78.51 ± 0.72% vs 78.06 ± 0.54%, 78.80 ± 0.88%, P = 0.654) between the normal weight group, the overweight group, and the obesity group **(**Table 3). In obese patients with asthma, FEV_1_% predicted (66.03 ± 10.30%), FVC% predicted (82.13 ± 8.11%), and FEV_1_/FVC ratio (62.42 ± 7.70%) were lower than those of normal weight patients with asthma, but the difference was not statistically significant.

**Table 3:** Adjusted means of forced expiratory volume in one second (FEV_1_), forced vital capacity (FVC), and FEV_1_/FVC according to the obesity in Korean boys and girls aged 10–18 years (*n* = 763).

	Normal weight	Overweight	Obesity	*P*
**All Participants**	*n* = 304	*n* = 235	*n* = 224	
FEV_1_ (%)	93.40 ± 1.21	93.77 ± 0.90	91.97 ± 1.47	0.529
FVC (%)	94.36 ± 1.04	94.42 ± 0.77	91.51 ± 1.26	0.125
FEV_1_/FVC (%)	78.51 ± 0.72	78.06 ± 0.54	78.80 ± 0.88	0.654
**No asthma**	*n* = 299	*n* = 228	*n* = 214	
FEV1 (%)	93.52 ± 1.21	94.05 ± 0.90	92.46 ± 1.49	0.575
FVC (%)	94.39 ± 1.05	94.38 ± 0.79	91.69 ± 1.30	0.181
FEV_1_/FVC (%)	78.60 ± 0.72	78.33 ± 0.53	79.04 ± 0.89	0.727
**Asthma**	*n* = 5	*n* = 7	*n* = 10	
FEV_1_ (%)	107.69 ± 11.97	91.48 ± 8.92	66.03 ± 10.30	0.156
FVC (%)	101.02 ± 9.43	97.06 ± 7.03	82.13 ± 8.11	0.511
FEV_1_/FVC (%)	86.77 ± 8.95	75.10 ± 6.67	62.42 ± 7.70	0.261

The data are shown as the means ± SEs (standard errors).

MetS, metabolic syndrome; FEV_1_, forced expiratory volume in one second; FVC, forced vital capacity. FEV_1_, FVC, and FEV_1_/FVC were adjusted for age, gender, square of height (m^2^), household income, alcohol drinking, smoking, residence, physical activity and atopic dermatitis. Statistical significance was determined using an analysis of covariance (ANCOVA).

### Metabolic syndrome and pulmonary function tests

After adjustment for age, sex, square of height (m^2^), weight (kg), household income, alcohol drinking, smoking, physical activity, and diagnosis of atopic dermatitis, subjects with MetS showed a significantly lower FEV_1_ predicted (91.54 ± 0.74% vs 94.64 ± 0.73%, P = 0.004), FVC% predicted (91.86 ± 0.63% vs 95.20 ± 0.63%, P < 0.001), and lower FEV_1_/FVC ratio (76.76 ± 0.43% vs 80.13 ± 0.43%, P < 0.001) than those without MetS (Table 4). In the no-asthma group, subjects with MetS had significantly lower FEV_1_% predicted (92.02 ± 0.73% vs 94.71 ± 0.73%, P = 0.013), lower FVC% predicted (92.01 ± 0.64% vs 95.18 ± 0.63%, P = 0.001), and lower FEV_1_/FVC ratio (77.03 ± 0.43% vs 80.23 ± 0.43%, P < 0.001) than those without MetS. Among subjects with or without MetS, those with self-reported asthma showed lower FEV_1_ predicted (77.12 ± 5.34% vs 92.96 ± 6.76%, *P* = 0.130), FVC% predicted (87.95 ± 3.98% vs 95.83 ± 5.04%, *P* = 0.299), and FEV_1_/FVC ratio (69.03 ± 12.74% vs 76.25 ± 9.81%, *P* = 0.277), but there were no statistically significant difference (Table 4).

**Table 4:** Adjusted means of forced expiratory volume in one second (FEV_1_), forced vital capacity (FVC), and FEV_1_/FVC according to the presence of metabolic syndrome (MetS) in Korean boys and girls aged 10–18 years (*n* = 763).

	No MetS	MetS	*P*
**All Participants**	*n* = 383	*n* = 380	
FEV_1_ (%)	94.64 ± 0.73	91.54 ± 0.74	0.004
FVC (%)	95.20 ± 0.63	91.86 ± 0.63	<0.001
FEV_1_/FVC (%)	80.13 ± 0.43	76.76 ± 0.43	<0.001
**No asthma**	*n* = 374	*n* = 367	
FEV_1_ (%)	94.71 ± 0.73	92.02 ± 0.73	0.013
FVC (%)	95.18 ± 0.63	92.01 ± 0.64	0.001
FEV_1_/FVC (%)	80.23 ± 0.43	77.03 ± 0.43	<0.001
**Asthma**	*n* = 9	*n* = 13	
FEV_1_ (%)	92.96 ± 6.76	77.12 ± 5.34	0.130
FVC (%)	95.83 ± 5.04	87.95 ± 3.98	0.299
FEV_1_/FVC (%)	76.25 ± 9.81	69.03 ± 12.74	0.277

The data are shown as the means ± SEs (standard errors).

MetS, metabolic syndrome; FEV_1_, forced expiratory volume in one second; FVC, forced vital capacity. FEV_1_, FVC, and FEV_1_/FVC were adjusted for age, gender, square of height (m^2^), weight (kg), household income, alcohol drinking, smoking, residence, physical activity and atopic dermatitis. Statistical significance was determined using an analysis of covariance (ANCOVA).

### Metabolic syndrome components and FEV_1_/FVC ratio

The results on the association between MetS components and FEV_1_/FVC ratio are presented in Table 5. MetS components including elevated WC, elevated SBP, elevated fasting glucose, and low HDL-C were independently associated with lower FEV_1_/FVC ratio (β coefficient; −0.243, −0.160, −0.111, and −0.088, respectively P < 0.05) after adjusting for age, sex, square of height (m^2^), weight (kg), household income, alcohol drinking, smoking, residence, physical activity, and atopic dermatitis. The direction and significance of all results for the non-asthmatic subjects remained the same with those of the overall cohort, whereas asthmatics subjects (n = 22) showed only elevated WC, and this was significantly related with lower FEV_1_/FVC ratio (β coefficient; −0.602, *P* = 0.047). In the normal weight group, MetS components including elevated WC, elevated SBP, and elevated fasting glucose were independently associated with lower FEV_1_/FVC ratio (β coefficient; −0.230, −0.196, and −0.142, respectively, P < 0.05) after adjusting for age, sex, square of height (m^2^), household income, alcohol drinking, smoking, residence, physical activity, and atopic dermatitis. The direction and significance of all results for the overweight/obese subjects were similar with those of normal weight subjects.

**Table 5:** Relationship between metabolic syndrome and it component and FEV_1_/FVC in Korean boys and girls aged 10–18 years according to asthma status and obesity (*n* = 763).

	Elevated WC	*P*	Elevated BP	*P*	Elevated glucose	*P*	Elevated TG	*P*	Reduced HDL-C	*P*	MetS	*P*
All participants (*n* = 763)	−0.243	<0.001	−0.160	<0.001	−0.111	0.002	−0.033	0.370	−0.088	0.018	−0.205	<0.001
**The presence of Asthma**												
No-asthmatics (*n* = 741)	−0.238	<0.001	−0.160	<0.001	−0.090	0.015	−0.040	0.295	−0.102	0.006	−0.199	<0.001
Asthmatics (*n* = 22)	−0.602	0.047	−0.102	0.701	−0.552	0.038	0.146	0.604	0.603	0.098	−0.350	0.277
**BMI criteria**												
Normal weight (*n* = 304)	−0.230	<0.001	−0.196	0.001	−0.142	0.015	0.042	0.479	−0.082	0.169	−0.168	0.006
Overweight/obese (*n* = 459)	−0.191	<0.001	−0.112	0.017	−0.098	0.035	−0.086	0.067	−0.087	0.066	−0.210	<0.001

Statistical significance was determined using an analysis of multivariate linear regression analyses after adjustment for age, gender, square of height (m^2^), weight (kg), household income, alcohol drinking, smoking, residence, physical activity and atopic dermatitis. WC, waist circumference; BP, blood pressure; TG, triglyceride; HDL-C, high-density lipoprotein cholesterol; MetS, metabolic syndrome.

## Discussion

This study showed that obesity was not significantly associated with pulmonary function but having MetS was significantly related with lower pulmonary function in a nationally representative population of children and adolescents in Korea. MetS was associated with lower lung function, regardless of obesity status, even in non-asthmatics. Among MetS components, WC was the most significant factor, and high SBP, high fasting glucose, and low HDL-C were significantly associated with lower FEV_1_/FVC ratio.

The relationship between obesity and pulmonary function in children and adolescent is controversial. In our study, there were no significant differences in FEV_1,_ FVC, and FEV_1_/FVC ratio between the normal, the overweight, and the obesity groups. Some studies also demonstrated no difference in FEV_1_, FVC, and FEV_1_/FVC ratio between obese/overweight and normal weight children^12,15^. However, in majority of studies, the lung functions of overweight/obese children were significantly associated with at least one of the spirometric variables, but the results are inconsistent^10,11,16^. A recent meta-analysis found more pronounced FEV_1_/FVC ratio deficit with unchanged FEV_1_ or FVC in obese children and lower FEV_1_, FVC, and total lung capacity in obese adults^16^. They reported that among obese subjects those with lower FEV_1_/FVC ratio showed greater lung capacity than those with relatively higher FEV_1_ and FVC, but the airway diameter has not grown proportionally^17–19^. Pulmonary function is lower in obese asthmatics^20,21^, but our study did not show any statistical relationship between obesity and pulmonary function. However, the FEV_1_ was considerably lower in obese asthmatic patients, and they showed obstructive lung pattern. Future research is needed to further clarify these findings.

There are few studies on the association between lung function and MetS in school-aged children, and the results are unclear in adults. In a Taiwanese study, restrictive lung function was significantly associated with MetS after adjusting for age, sex, BMI, alcohol use, smoking, and physical activity^14^. In a study of adolescents in the USA, MetS was related with lower FEV_1_/FVC ratio, and such association was more pronounced among asthma patients^22^. In our study, patients with MetS showed lower FEV_1,_ FVC, and FEV_1_/FVC ratio regardless of obesity and asthma status. Meanwhile, although asthma patients with MetS did not show any statistical significance, they showed substantially lower FEV_1_ and obstructive pattern of lung function. A By contrast, a previous study reported an inverse correlation between MetS and lung function in asthma patients^22^. This discrepancy might be due to the limited number of asthmatic subjects in this study and the use of self-reported asthma diagnosis. Further studies should be conducted to investigate the relationship between pulmonary function and MetS in subjects with asthma.

MetS is a complex disorder that includes chronic inflammation characterized by abdominal obesity, hyperglycemia, hypertension, and dyslipidemia. Previous studies have investigated the relationship between individual components of MetS and pulmonary function, but the influence of each factor seems to vary between each study group. For example, lower HDL-C was the major predictor for impaired pulmonary function in an Italian population^23^. Meanwhile, abdominal obesity and hyperglycemia were reported to be significant associated with pulmonary function impairment and MetS in a Japanese male population^24^. Another study that included obese patients in the Netherlands reported that only hypertension was the significant MetS component associated with lower FEV_1_/FVC ratio^25^. In our study, which was conducted in Korean children and adolescents, increased WC, BP, fasting glucose, and low HDL-C were significantly associated with lower FEV_1_/FVC ratio.

Previous studies suggested several potential hypotheses explain the relationship between MetS and impaired pulmonary function. One is that systemic inflammations caused by MetS are associated with altered pulmonary immune and oxidative stress reactions^26–28^. Another is that insulin resistance alters protein metabolism and results in epithelial damage^29^. Interestingly, MetS was also associated with lower pulmonary function in the normal weight group and the non-asthmatics. These findings provide additional evidence to support the hypothesis that aside from the classic atopic airway inflammation, other mechanisms are involved in lower pulmonary function in asthma patients. The range to which these mechanisms mediated the consequence of metabolic anomalies on pulmonary function needs further research. Moreover, future intervention studies will be needed to determine if treatment of MetS can prevent lower lung function in subjects with MetS.

This study has several limitations. First, this was a cross-sectional study that measured pulmonary function and MetS components at a single period rather than doing long-term repeated measurements. Therefore, prospective longitudinal studies are needed to identify the definite pathophysiologic mechanism by which MetS influences pulmonary function. Second, the sample designed for KNHANES was selected, but this study showed higher proportion of MetS than that previously reported^7,8^, indicating that there may have been a higher number of high-risk patients who are obese or have abnormal blood tests that participated in the pulmonary function tests. However, this was the first study in Korea to investigate the association between MetS and pulmonary function in a representative population of children and adolescents. The results will help to elucidate the pathogenesis of MetS and lung-related diseases.

## Conclusion

Pulmonary function was not significantly different according to obesity status among Korean children and adolescents. However, patients with MetS had significantly lower lung functions, and MetS components were independently associated with pulmonary function. Physicians should be aware of the adverse effects of MetS on lung function.

## Methods

### Study population

This study was conducted using data obtained from the 2009–2011 Korea National Health and Nutrition Examination Survey (KNHANES). The KNHANES is a cross-sectional, nationwide, and representative survey regularly conducted by the Chronic Disease Surveillance Department, Korean Centers for Disease Control and Prevention. The KNHANES enrolls approximately 10,000 people annually as a survey sample and collects information through health interviews, health examinations, and nutrition surveys. To capture a representative population, multi-stage stratified statistical design is applied in this survey^30^. All participants in the study provided written consent form. And the researcher obtained informed consent from a parent and/or legal guardian for study participation. This survey received institutional review board approval from the Korean Centers for Disease Control and Prevention (approval no: 2009-01CON-03-2C, 2010-02CON-21-C, 2011-02CON-06-C). Of the 17,720 individuals included in the KNHANES, we identified children and adolescents aged 10–18 years as potential candidates for this study. For users and researchers who promise to follow the research ethics throughout the world, micro-data (in the form of SAS and SPSS files) can be downloaded by e-mail and the details of KNHANES can be accessed on the KNHANES website in English version (http://knhanes.cdc.go.kr). All methods were performed in accordance with the relevant guidelines and regulations

### Measurements

Anthropometric measurements were obtained for all participants by trained staff. Height was measured to the nearest 0.1 cm with a portable stadiometer (Seca, Hamburg, Germany), while weight was measured to the nearest 0.1 kg on a medical balance scale (GL-6000-20; CAS, Seoul, Korea). The BMI was calculated as weight (kg) divided by height squared (m^2^). Height, weight, and BMI standard deviation score (SDS) was determined using the 2007 Korean reference data^31^. Waist circumference (WC) was measured at the midpoint between the lowest margin of the rib and the uppermost lateral border of the iliac crest during expiration using a flexible tape (Seca 225; Hamburg, Germany). The mean and standard deviation of anthropometric and body composition values (height, weight, BMI, and WC) were calculated. Age- and gender-specific percentiles were calculated by using the Lambda-MuSigma (LMS) method (LMS Chartmaker Pro, version 2.5, Medical Research Council, Cambridge, UK) to estimate the skewness (L), median (M), and coefficient of variation (S). The blood pressure (BP, mmHg) was measured from the right upper arm, after the subject had rested for 5 minutes, in a seated position using calibrated sphygmomanometer (Baumanometer Desk model 0320, Baum, NY, USA) and an appropriately sized cuff. Systolic blood pressure (SBP) and diastolic blood pressure (DBP) were measured three times, and the second and third measurements were averaged and used for the analysis.

A blood sample was collected from each participant after overnight fasting. The samples were analyzed using an auto-analyzer (Hitachi automatic analyzer 7600, Hitachi, Tokyo, Japan) to determine the serum levels of glucose, total cholesterol, triglyceride (TG), and high-density lipoprotein cholesterol (HDL-C). The level of low-density lipoprotein cholesterol (LDL-C) was calculated with Friedewald’s equation^32^. Pulmonary function was measured using a spirometer (model 2130; SensorMedics, CA, USA); all spirometric measurements were pre-bronchodilators. The FVC and FEV_1_ defined according to the American Thoracic Society guidelines, were recorded^33^. Spirometry is simple, noninvasive method used to monitor clinically important changes in lung functions. FVC is the maximum volume of air exhaled with maximum forced expiration effort as quickly as possible. FVC typically is decreased in restrictive pulmonary diseases. FEV_1_ is total volume of air exhaled during the first seconds with maximal effort. FEV_1_ is decreased in obstructive disorders. FEV_1_/FVC ratio is the percentage of the FVC expired in one second. If the FEV_1/_FVC ratio decreased, it suggests an obstructive defect. The FVC, FEV_1_, and FEV_1_/FVC ratio were measured and are expressed as both absolute values (L) and predicted values (%) calculated from large Korean healthy population^34^.

### Definition

Obesity was defined using the 2007 Pediatric Adolescent Standard Growth Chart from the Center for Disease Control and the Association of Korean Pediatrics^31^. Weight classifications according to the BMI diagnostic criteria were defined as normal weight (<85th percentile by sex and age; overweight (≥85th percentile and <95th percentile); and obesity (≥95th percentile or BMI ≥25 kg/m^2^)^3,35^. MetS was defined as the presence of at least three of the following five criteria in the modified NCEP-ATP III^5^: (1) elevated WC (i.e., ≥90th percentile for age and sex); (2) elevated BP (i.e., SBP or DBP ≥90th percentile for age and sex and height according to 2007 Korean reference data)^31^; (3) glucose intolerance (i.e., fasting glucose concentrations ≥110 mg/dL); (4) elevated TG (i.e., serum TG concentrations ≥110 mg/dL); and (5) low HDL-C (i.e., levels of serum HDL-C < 40 mg/dL). Asthma was defined as a self-assessment diagnosis in a health interview survey. The survey included questions about the use of asthma medication (including inhalers, aerosols, or tablets), experience of wheezing in the last 12 months, and asthma diagnosed by a doctor.

### Statistical analysis

Statistical analyses were performed using SPSS software for Windows (SPSS version 23.0, IBM SPSS Inc., Chicago, IL, USA). Normally distributed variables are presented as means ± standard errors (SE), while categorical variables are presented as percentages (%). Because height, weight, BMI, and WC were not evenly distributed among the different age groups, the SDS of these measures were determined using the LMS method according to the 2007 Korean National Growth Charts^31^. Statistical significance was determined for continuous variables using analysis of variance according to obesity or independent t-test according to the presence of MetS. Meanwhile, a chi-square test was used for categorical variables. Analysis of covariance was used to evaluate pulmonary function according to obesity or presence of MetS after adjusting for possible confounding factors, such as age, sex, square of height (m^2^), weight, household income, alcohol drinking, smoking, residence, physical activity, and atopic dermatitis. The data were presented as the adjusted means ± SE. Additional analysis was also performed depending on the presence or absence of asthma. To investigate the associations between pulmonary function and MetS and its components, multiple linear regression analyses were conducted after adjustment for age, sex, square of height (m^2^), weight, household income, alcohol drinking, smoking, residence, physical activity, and atopic dermatitis among all participants. Additionally, subgroup analysis was performed because the relationship could change depending on the patient’s characteristics. Therefore, subgroups were analyzed by the presence of asthma and normal weight or overweight/obesity. Corresponding standardized regression coefficients (β) were determined, and *P* values of < 0.05 were considered statistically significant.

## Data Availability

All the data generated and/or analyzed during the current study are included in this article and are available from the corresponding author on reasonable request.
